# Dolutegravir use is related to lower HTLV-1 proviral load in people co-infected by HIV-1

**DOI:** 10.1038/s43856-025-01312-9

**Published:** 2025-12-18

**Authors:** Tatiana Fernandez, María B. Arriaga, Rafaela Mayoral, Eduardo M. Netto, Carlos Brites

**Affiliations:** 1 Laboratório de Pesquisa em Infectologia (LAPI), Hospital Universitário Professor Edgard Santos, Universidade Federal da Bahia, Salvador, Brazil; 2 School of Medicine, Department of Medicine, Universidade Federal da Bahia, Salvador, Brazil

**Keywords:** Viral infection, Infectious diseases

## Abstract

**Background:**

Infection by human T-cell lymphotropic virus type 1 (HTLV-1) affects millions of people worldwide and causes severe diseases. To date, no specific treatment is available for HTLV infection. The purpose of this study was to determine the impact of Dolutegravir use in reducing HTLV-1 proviral load (PVL) in HIV-HTLV1 coinfected subjects on stable antiretroviral therapy.

**Methods:**

In this cross-sectional study we quantified HTLV-1 PVL in HIV-HTLV1 coinfected patients and HTLV-1 mono-infected ones. We compared HTLV-1 proviral load across groups, adjusting for age and sex, antiretroviral therapy use and CD4/CD8 cells count. We used a propensity score derived from a regression model for Dolutegravir use and HTLV-1 PVL, that included covariates like age and antiretroviral therapy duration.

**Results:**

Eighty-eight patients were included, 44 coinfected by HIV and HTLV-1 and 44 controls. Linear regression shows an association between Dolutegravir use and lower HTLV-1 proviral load values (p = 0.042). Participants using Dolutegravir are significantly more likely to have an HTLV-1 proviral load below the median (205 DNA copies/mm^3^) than those antiretroviral therapy -naïve (p = 0.003). In logistic regression, Dolutegravir use is significantly associated with undetectable HTLV-1 proviral load (<50 DNA copies/mm^3^, p = 0.015), and HTLV-1 proviral load lower than quartile 75 values (945 DNA copies/mm^3^, p = 0.021).

**Conclusions:**

Dolutegravir use is consistently associated with lower HTLV-1 proviral load in HIV-HTLV-1 coinfected patients. This indicates that Dolutegravir may be an effective treatment for HTLV-1 infection.

## Plain language summary

Human T-cell lymphotropic virus type 1 (HTLV-1) may lead to serious illness, yet there is no specific treatment. Dolutegravir is a medication that is used to treat people with human immunodeficiency virus (HIV). The aim of this study was to determine if Dolutegravir use could reduce HTLV-1 levels in patients who are coinfected with both HIV and HTLV-1 and receiving treatment for HIV. We analyzed the HTLV-1 levels of 44 people who were coinfected with HIV-HTLV-1 who were receiving Dolutegravir, and 44 people infected with HTLV-1 only (not receiving Dolutegravir). We demonstrated that Dolutegravir use is associated with lower HTLV-1 levels. Dolutegravir use may potentially be an effective treatment for HTLV-1 infection.

## Abstract (web summary)

Fernandez et al. compare the HTLV-1 proviral load in people with HIV-1-HTLV-1 coinfection on stable antiretroviral therapy or people with HTLV-1 single infection without antiretroviral therapy. Dolutegravir use for longer than six months is associated with lower HTLV-1 proviral load.

## Introduction

Human T-cell lymphotropic virus (HTLV) infects ~10–20 million people worldwide^1,2^ with an estimated 5–10% of these individuals expected to develop a spectrum of diseases, including adult T-cell leukemia/lymphoma (ATL), HTLV-1-associated myelopathy/tropical spastic paraparesis (HAM/TSP), and other inflammatory conditions^2–4^. Despite decades of research, there is currently no effective antiviral therapy to target HTLV-1, and treatment options for HTLV-1 associated diseases remain limited and largely supportive. HTLV-1 infection typically progresses to clinical disease over decades, requiring a long follow up time for interventional studies. Additionally, the lack of reliable biomarkers to assess disease progression poses a challenge for evaluating treatment strategies targeting the virus.

Unlike HIV, which demonstrates high replicative activity and viral turnover, HTLV-1 predominantly maintains persistent infection through clonal expansion of infected cells^5,6^. This difference in replication strategy has posed significant challenges for the development of antiretroviral therapies for HTLV-1. Moreover, the proviral load—the integrated viral DNA within host cells—has been strongly correlated with disease progression in HTLV-1-infected individuals, underscoring the need for interventions capable of reducing proviral burden. Higher HTLV proviral load (PVL) is predictive for development of HTLV-associated diseases^7–9^.

Antiretroviral therapy (ART) has been a cornerstone in managing HIV infection, dramatically improving patient outcomes and reducing viral loads to undetectable levels. Emerging evidence suggests that contemporary ART regimens may also influence the proviral load of HTLV infections^10,11^. Even though the available evidence on this point is still limited, the potential reduction in HTLV PVL following ART initiation is a promising finding, suggesting that ART might be an effective strategy for mitigating the impact of HTLV in coinfected individuals.

The integrase enzymes of HIV and HTLV-1 share some structural and

functional similarities, and some clinical studies tested raltegravir, the first integrase inhibitor used in antiretroviral therapy, to treat HTLV infection^11–13^. Dolutegravir (DTG), a second-generation integrase strand transfer inhibitor (INSTI), has become a cornerstone in the management of HIV infection due to its potent antiviral efficacy, high genetic barrier to resistance, and favorable safety profile^14^. Dolutegravir is the first-line ART drug in Brazil^15^. As HIV and HTLV share similar transmission routes, coinfection with these two viruses is often detected in endemic areas. In Bahia, at least 1% of people living with HIV (PLHIV) are coinfected by HTLV-1 and are on stable antiretroviral therapy (ART), making this an ideal population to evaluate the effect of this drug on HTLV-1 PVL.

Here, we present the results of a clinical investigation exploring the impact of dolutegravir on HTLV-1 proviral load in individuals co-infected with HIV and HTLV-1. Our findings demonstrate that dolutegravir use can reduce HTLV-1 proviral loads in people living with HIV-1-HTLV-1 coinfection. To the best of our knowledge, this study is the first to demonstrate a significant reduction in HTLV-1 proviral load associated with antiretroviral therapy.

## Methods

### Participants and setting

This is a cross-sectional study conducted at Professor Edgard Santos Hospital Complex (C-HUPES), a main referral center for HIV and HTLV care in Salvador, Bahia, Brazil.

The study included consecutive PLHIV with a Western blot-confirmed HTLV-1 coinfection, aged 18 years or older. Exclusion criteria were HTLV-2 infection, active malignancies, and severe chronic liver disease caused by hepatitis B or C viruses. Additionally, a group of HTLV-1 mono-infected individuals, unexposed to ART, was selected as controls. All participants underwent HTLV-1 PVL quantification, as well as CD4 and CD8 measurement. Sociodemographic data and information about duration of HIV and HTLV infections, ART regimen and time on use, HIV viral load (for coinfected subjects), and HTLV-related diseases were collected through interviews, and medical record reviews. All patients with HIV/HTLV-1 coinfection who received care during the study period were invited to participate in the study.

### Laboratory procedures

Samples of whole blood were collected, and DNA extraction was performed from peripheral blood leukocytes using the PureLink Genomic DNA Mini Kit (Invitrogen, Massachusetts, USA), following the manufacturer’s instructions.

The HTLV-1 proviral load was measured by real-time polymerase chain reaction (qPCR) based on a previously published study, employing the TaqMan® system on the ABI PRISM 7000 platform (Applied Biosystems, Foster City, CA)^16^. The assay targeted three regions: the albumin gene as an endogenous control, and nonhomologous regions of the pol gene of HTLV-1 and HTLV-2. Primers and probes were designed and synthesized via the Assay-by-Design SM service (Part Number 4331348), using the primer sequences CAACCCCACCAGCTCAGG and GGGAAGGTTAGGACAGTCTAGTAGATA.

Initially, relative quantification was performed based on the ratio between the target and the endogenous control. The final equation for absolute quantification of HTLV-1 proviral load incorporates the leukocyte count per mm³, and results are expressed in DNA copies /mm^3^ of whole blood.

### Statistical analysis

Categorical variables were presented as frequency and percentages and compared using a two-sided Chi-square test. The Gaussian distribution of continuous variables was assessed using the Kolmogorov-Smirnov test (no variable was normally distributed) and were displayed as median and interquartile range (IQR) and compared using the Mann-Whitney *U* test. “DTG use” was defined as continuous use of the medication for at least 6 months. HTLV-1 PVL was categorized based on median cut-off values (≤205 DNA copies/mm³ and >205 DNA copies/mm³), IQR 75 (≤945 DNA copies/mm³ and >945 DNA copies/mm³) and undetectable (≤50 DNA copies/mm³) or detectable (>50 DNA copies/mm³) categories.

In this study, propensity score (PS) was used to minimize the influence of confounding factors between the comparison study groups. Associations between the use of DTG and HTLV-1 PVL were tested using PS adjusted logistic regression models^17^. The PS was derived from a regression model for DTG use and calculated for each participant incorporating covariates such as age (in years), duration of ART use and CD4+ values. This PS was then adjusted for the exposure variable “use of DTG” in four main outcome models: (i) HTLV-1 PVL (linear regression), (ii) HTLV-1 PVL > 205 DNA copies/m2m³ (median), (iii) >945 DNA copies/mm³ and (iv) >50 DNA copies/mm³. We used bootstrap resampling −1000 samples- for internal validation. Results were expressed as odds ratios and 95% confidence intervals (CIs). P < 0.05 (two-sided) were considered statistically significant.

### Ethical statement

The study was conducted in accordance with the Declaration of Helsinki and was approved by the Research Ethics Committee of the Faculty of Medicine of the Federal University of Bahia (n° 1.035.826) and the Research Ethics Committee of the Maternidade Climério de Oliveira (n° 4.643.372). All participants provided a written informed consent.

## Results

### Participants’ characteristics

**Fig. 1:**
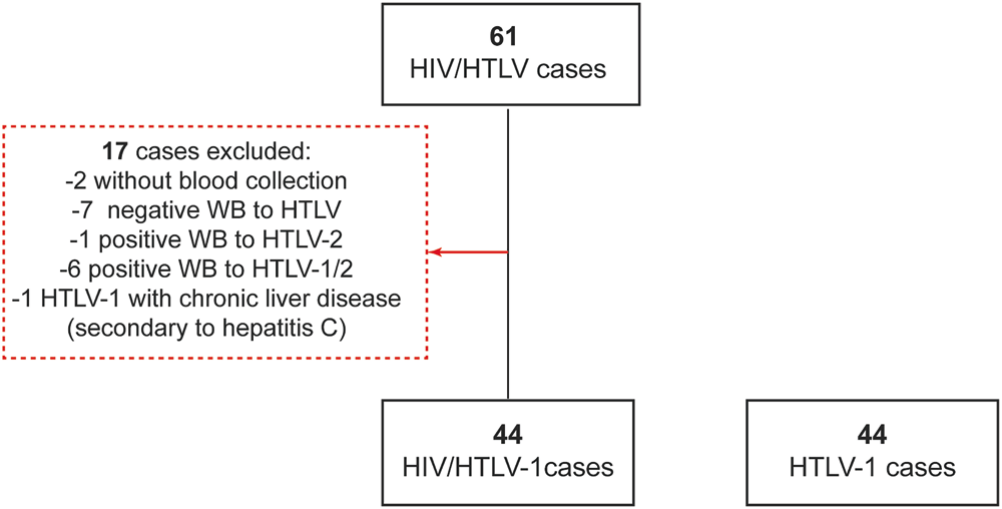
Flowchart of study. Abbreviations: HTLV-1 human T cell leukemia virus type I, WB western blot.

Between 2021 and 2023, 61 coinfected individuals were invited to participate in the study. Of these, 2 participants were excluded because they did not attend the sample collection visit; 7 were excluded due to negative WB results for HTLV; 1 participant was excluded due to a positive WB result for HTLV-2; and 6 participants were excluded due to positive WB results for HTLV-1 and HTLV-2. Additionally, 1 participant with a positive WB result for HTLV-1 was excluded due to severe chronic liver disease secondary to hepatitis C virus (Fig. 1). Forty-four individuals living with HIV/HTLV-1 coinfection (33 asymptomatic carriers and 11 HTLV-1-related diseases) were included. The control group consisted of 44 HTLV-1 mono-infected participants (30 asymptomatic carriers, and 14 presenting with HTLV-1-related diseases).

### Use of ART and impact on HTLV-1 proviral load

As expected, HTLV-1 mono infected participants were not on ART. In contrast, 39 (88.6%) of coinfected participants received ART, 26 (59.1%) of them on DTG, as part of their HIV ongoing care (Table 1). Table 2 displays a list of all ART regimens used by study participants coinfected by HIV and HTLV-1. Coinfected individuals were also older compared to mono infected ones (median age: 57 vs 50 years; p = 0.002). In addition, the median CD4+ cell count and CD4+/CD8+ ratio were significantly lower among coinfected participants (p < 0.001). Median PVL was only marginally different across groups (p = 0.057). Detailed participant characteristics are presented in Table 1. However, a direct comparison between coinfected participants using DTG (excluding those ART-naïve or who were using other regimens), showed this group had a median PVL significantly lower (105 DNA copies/mm^3^) than that observed in mono infected ones (277 DNA copies/mm^3^, p = 0.047). Median PVL of coinfected participants on DTG (91 DNA copies/mm³, IQR: 50-415 DNA copies/mm³) was lower than that found in participants using other ART regimens (270 DNA copies/mm³, IQR: 50-1027 DNA copies/mm³) but the difference did not reach statistical significance, due to the small sample size (calculated power below 20%).

**Table 1:** Characteristics of the study population by infection status

Characteristics	HTLV/HIV coinfection (n = 44)	HTLV-1 monoinfection (n = 44)	p-value
**Median (IQR) age (years)**	57 (51–62)	50 (45–56)	0.002
**Female, n (%)**	27 (61.4)	32 (72.7)	0.365
**Race n (%)**^**a**^ **Pardo/Black**	43 (100)	43 (100)	NA
Schooling, n (%)			0.001
Illiterate or incomplete basic	23 (52.3)	13 (29.5)	
Complete basic	11 (25.0)	3 (6.8)	
High school or college education	10 (22.7)	28 (63.6)	
**ART use, n (%)**^**b**^	39 (88.6)	0 (0.0)	NA
**DTG use (%)**	26 (59.1)	0 (0.0)	NA
**Median (IQR) Proviral load (DNA copies/mm³)**	125 (50–552)	277 (50–1487)	0.057
**Median (IQR) CD4+ cells count**	588 (491–1082)	1361 (1039–1580)	0.000
**Median (IQR) CD8+ cells count**	797 (545–1130)	588 (489–772)	0.010
**Median (IQR) ratio CD4 + /CD8 + cells count**	0.88 (0.47–1.47)	2.20 (1.64–2.68)	0.000

Categorical variables were compared by Chi-square test. Continuous variables were compared using the Mann-Whitney *U* test. All tests were two-sided.

Two participants who self-identified as white were excluded from this analysis.

*HTLV-1* human T cell leukemia virus type I, *IQR* interquartile range, *ART* antiretroviral therapy, *DTG* Dolutegravir, *NA* not applicable.

^a^Race category was self-declared.

^b^ART use category in addition to DTG, the participants used protease inhibitor, Zidovudina or Efavirenz.

**Table 2:** Antiretroviral Therapy (ART) used by participants

Antiretroviral Therapy (ART)	n (%)
All	39 (100)
ATV/r+TDF+3TC	3 (7.6)
AZT+NVP+3TC	2 (5.1)
DRV/r+TDF+3TC	1 (2.6)
DTG+3TC	9 (23)
DTG+ABC+TDF+3TC	1 (2.6)
DTG+ATV/r	1 (2.6)
DTG+ATV/r+TDF+3TC	1 (2.6)
DTG+AZT+3TC	1 (2.6)
DTG+DRV/r+3TC	1 (2.6)
DTG+TDF+3TC	15 (38.5)
EFV+AZT+3TC	1 (2.6)
EFV+TDF+3TC	3 (7.6)

*ATV/r* Atazanavir/ritonavir, *TDF* Tenofovir Disoproxil Fumarate, *3TC* Lamivudine, *AZT* Azidothymidine, *NVP* Nevirapine, *DRV/r* Darunavir/ritonavir, *DTG* Dolutegravir, *ABC* Abacavir, *EFV* Efavirenz.

On the other hand, median PVL for participants on DTG (91 DNA copies/mm³, IQR: 50-415 DNA copies/mm³) was significantly lower than that detected for those infected by HTLV-1 only (277 DNA copies/mm³, IQR: 50-1487 DNA copies/mm³), while the comparison between participants using non-DTG regimens and monoinfected ones was similar (270 DNA copies/mm³, IQR: 50–1027 DNA copies/mm³ vs. 277 DNA copies/mm³, IQR: 50-1487 DNA copies/mm³, p = 0.513).

Table 3 shows characteristics of participants according to the median PVL. It was observed that CD4+ cell counts were associated with higher levels of PVL for HTLV-1 (p = 0.007). CD4+/CD8+ cell counts were missing in 4 participants in the HTLV-1 group and 1 in the coinfected group. In addition, median PVL for HTLV-1 was associated with lower median age (p < 0.001), or ART use (p = 0.018). The use of DTG-based ART was significantly associated with lower median PVL for HTLV-1 (p = 0.005). Median PVL was not associated with HTLV-1-related diseases in the univariate analysis, nor with HIV plasma viral load (r^2^ = 0.296, p = 0.051).

**Table 3:** Characteristics of the study population by median HTLV-1 Proviral load (PVL)

Main characteristics of study population	All	HTLV-1 PVL > 205 DNA copies/mm³	HTLV-1 PVL ≤ 205 DNA copies/mm³	p -value
	n = 88	(n = 44)	(n = 44)	
**Median (IQR) age (years)**	54 (47–59)	50 (44–56)	57 (52–62)	0.000
**Sex, n (%)**	88 (100)			0.112
Female	59 (67)	33 (75)	26 (59.1)	
Male	29 (33)	11 (25)	18 (40.9)	
**Race, n (%)**^a^	86 (98)			NA
Black/Pardo	86 (98)	44 (100)	42 (100)	
**Schooling, n (%)**	88 (100)			
Illiterate or incomplete basic	36 (41)	19 (43.2)	17 (38.6)	0.664
Complete basic	14 (16)	7 (15.9)	7 (15.9)	
High school or college education	38 (43)	18 (40.9)	20 (45.5)	
**ART use, n (%)**^b^	88(100)			0.018
Yes	39 (44.3)	14 (31.8)	25 (56.8)	
No	49 (55.7)	30 (68.2)	19 (43.2)	
**DTG use, n (%)**	88 (100)			0.005
Yes	26 (29.5)	7 (15.9)	19 (43.2)	
No	62 (70.5)	37 (84.1)	25 (56.8)	
**Median (IQR) CD4+ cells count**	1025 (567–1375)	1198 (804–1564)	768 (520–1136)	0.007
**Median (IQR) CD8+ cells count**	644 (513–863)	638 (513–863)	688 (519–846)	0.953
**Presence of HTLVrelated disease, n (%)**	88 (100)			0.813
Yes	25 (28.4)	12 (27.3)	13 (29.5)	
No	63 (71.6)	32 (72.7)	31 (70.5)	

Categorical variables were compared by Chi-square test. Continuous variables were compared using the Mann-Whitney *U* test. All tests were two-sided.

^a^Race category was self-declared. Two participants who self-identified as white were excluded from this analysis.

^b^ART use category in addition to DTG, the participants used protease inhibitors, or Efavirenz.

### Association between use of Dolutegravir and proviral load levels

**Fig. 2:**
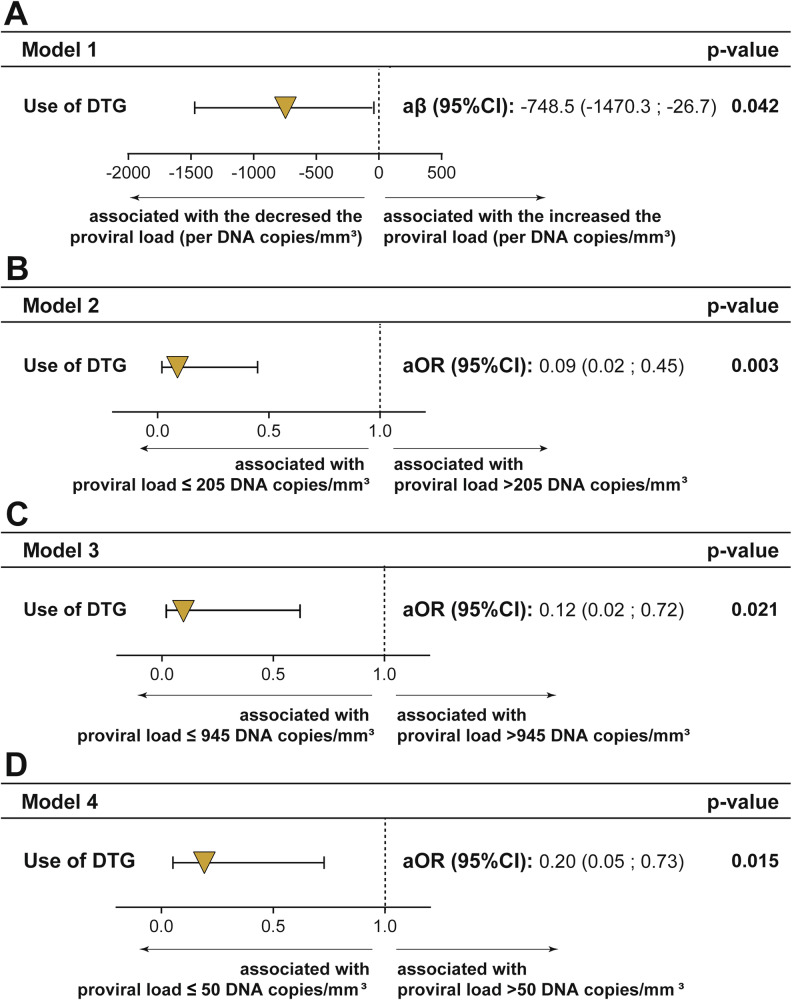
Logistic regressions were used to test associations between use of. DTG and proviral load. **A** increasing HTLV-1 PVL **B** HTLV-1 PVL > 205 DNA copies/mm^3^, **C** HTLV-1 PVL > 945 DNA copies/mm^3^ and **D** HTLV-1 PVL > 50 DNA copies/mm^3^. The propensity score was used as a covariate in each model. Two-sided confidence intervals of the adjusted Odds Ratio (aOR) and the adjusted coefficient (aβ) were calculated. Variables used to calculate the propensity score: age, time of use of antiretroviral therapy and CD4 + . Abbreviations: DTG Dolutegravir, CI confidence interval.

Four logistic regression models were used to test the impact of DTG use on HTLV-1 PVL, adjusted with the estimated propensity score (Fig. 2). The balancing score before and after adjusting for the propensity of use of DTG is displayed in the Supplementary Table 1.

Model 1 (Fig. 2A), thorough linear regression, shows that DTG was significantly associated with lower HTLV-1 PVL values (β coefficient: −748.5, 95%CI: −1470.3; −26.7, p = 0.042). Model 2 (Fig. 2B) and Model 3 (Fig. 2C) showed that the use of DTG was associated with a HTLV-1 PVL ≤ 205 DNA copies/mm³ [adjusted Odds ratio (aOR): 0.09, 95% CI: 0.02; 0.45, p = 0.003] and HTLV-1 PVL ≤ 945 DNA copies/mm³ (aOR: 0.12, 95% CI: 0.02; 0.72, p = 0.021), respectively. Finally, Model 4 (Fig. 2D) shows a significant association between DTG use and HTLV-1 PVL values < 50 DNA copies/mm³ (aOR: 0.20, 95% CI: 0.05; 0.73, p = 0.015) values.

Likewise, when we performed the internal validation for each model using the bootstrapping technique, the use of DTG was significantly associated with lower HTLV-1 PVL values (Supplementary Table 2), HTLV-1 PVL ≤ 205 DNA copies/mm³ (Supplementary Table 3), ≤945 DNA copies/mm³ (Supplementary Table 4) and ≤50 DNA copies/mm³ (Supplementary Table 5).

## Discussion

DTG is a widely used integrase inhibitor and recommended as first- and second-line treatment for HIV by the World Health Organization^18^. In our study 66.7% of ART users were on a DTG-based regimen. Use of DTG was significantly associated with decreased HTLV-1 PVL, undetectable HTLV-1 PVL, and with a HTLV1 PVL lower than the median or 75% IQR values. As observed in PLHIV, DTG was well tolerated and the potent inhibition of HTLV-1 integrase observed in previous in vitro studies^19^ was confirmed, in vivo, by our findings.

Although lamivudine (3TC), tenofovir (TDF) and zidovudine (ZDV) were tested against HTLV infection, only TDF or ZDV demonstrated a limited efficacy in inhibiting HTLV, but failed in decreasing proviral load in a few, small sample trials^20^. However, ZDV is no longer a recommended drug for treatment of HIV infection. Among protease inhibitors only darunavir showed an in vitro weak antiviral effect against HTLV, and non-nucleoside reverse transcriptase inhibitors are also ineffective against that virus, as NNRTIs do not bind effectively to the HTLV-1 RT active site^21–23^. Thus, it was not expected that any of the drugs in previous antiretroviral regimens have an efficacy against HTLV infection capable of providing a sustainable decrease in HTLV proviral load. According to the Brazilian Ministry of Health, currently around. 80% of patients on antiretroviral treatment are using DTG (plus either 3TC or TDF/3TC) as the base of contemporary ART regimen.

There are no previous studies in literature involving the use of DTG in people living with HTLV-1. Among the integrase inhibitors, only raltegravir was tested in humans, but without any significant impact observed on HTLV-1 PVL. However, raltegravir studies were small and of short duration^11,12^. Our study evaluated HIV-1HTLV-1 coinfected subjects on ART use for a longer time, which allowed us to detect the impact of DTG use on HTLV-1 PVL. The implications of such findings are substantial for clinical practice and research. If ART is proven to be effective in reducing HTLV proviral loads, it could lead to new therapeutic strategies for managing HTLV-associated conditions. It could also influence treatment guidelines for coinfected patients, highlighting the need for a comprehensive approach that addresses both HIV and HTLV.

In addition to the obvious conclusion on the potential use of ART for HTLV1 infection treatment, our study also challenges the existing paradigm on the low relevance of HTLV-1 active replication, due to predominantly clonal viral expansion during the infection. The observed impact of ART on HTLV-1 PVL indicates an important role of active replication of HTLV-1. This finding opens the perspective of antiviral treatment and prevention with ART, as occurs in HIV-1 infection.

Our study had several limitations, such as the small number of participants on ART and the absence of immune activation markers, that could influence HTLV PVL. In addition, we cannot rule out the potential impact of other ART components on the results. Furthermore, the ideal control group should include exclusively asymptomatic people, as the presence of HTLV-1-associated diseases could influence HTLV-1 PVL. However, there was no significant difference between HTLV1 PVL among symptomatic subjects with or without HIV infection, in the current study. In addition, there is a lack of studies with larger samples, making our study one of the largest already conducted to assess the effects of ART on HTLV-1 infection. Participants on DTG-based regimens (but not on non-DTG regimens) had a significantly lower HTLV PVL than monoinfected ones.

In conclusion, the evidence supporting the effect of antiretroviral drugs in decreasing HTLV proviral load in HIV-HTLV coinfected subjects can be a significant advance in the management of HTLV-1 infection. This finding provides a basis for future research aimed at optimizing treatment strategies for coinfected patients. The potential benefits of ART in controlling HTLV highlight a critical area of investigation with profound implications for patient care and treatment outcomes. Large, prospective, controlled studies are needed to confirm the role of ART on the management of HTLV-1 infection.

## Supplementary information

Supplementary Information

Description of Additional Supplementary Files

Supplementary Data 1

## Data Availability

Source data are provided for all results presented in the main manuscript and available as Supplementary Data 1.
