# Supplementary material for: Dolutegravir use is related to lower HTLV-1 proviral load in people co-infected by HIV-1
Source: Commun Med (Lond). 2025 Dec 18;6:54. doi: 10.1038/s43856-025-01312-9 (PMC12830689; doi:10.1038/s43856-025-01312-9)
Supplement: Supplementary file 1 — Supplementary Information [file 43856_2025_1312_MOESM1_ESM.pdf]

Supplementary Material

Supplemental Table 1.

Supplemental Table 2.

Supplemental Table 3.

Supplemental Table 4.

Supplemental Table 5.

Supplemental Table 1. Covariate balanced before and after adjusting for the propensity of use of dolutegravir

	Use of DTG	
**Characteristic**	**unadjusted OR (95%CI)**	**PS-adjusted OR (95%CI)**
**Age**	1.07 (1.01-1.12)	1.05 (1.0-1.1)
**ART use time**	1.02 (1.01-1.02)	1.01 (1.0-1.02)
**CD4+**	0.998 (0.997-0.999)	0.998 (0.997-1)

PS: propensity score, DTG: Dolutegravir, OR: odds ratio, CI: confidence interval

Supplemental Table 2. Linear regression using bootstrap resampling was performed between use of DTG and proviral load among coinfected individuals with HIV-HTLV-1

Parameter	adjusted β	95% (confidence interval)	p-value
**Use of DTG**	-748.53	1132.5 - 322.08	0.004

Propensity score was used as a covariate in the model.
Variables used to calculate the propensity score: age, time of use of antiretroviral therapy and CD4+.

Bootstrap results are based on 1000 bootstrap samples

DTG: Dolutegravir, OR: odds ratio

Supplemental Table 3. Logistic regression using bootstrap resampling was performed to tested association between use of DTG and proviral load >205 DNA copies/mm^3 among coinfected individuals with HIV-HTLV-1

Parameter	adjusted OR	95% (confidence interval)	p-value
**Use of DTG**	0.09	0.01 - 0.33	0.002

Propensity score was used as a covariate in the model.
Variables used to calculate the propensity score: age, time of use of antiretroviral therapy and CD4+.

Bootstrap results are based on 1000 bootstrap samples

DTG: Dolutegravir, OR: odds ratio

Supplemental Table 4. Logistic regression using bootstrap resampling was performed to tested association between use of DTG and proviral load >945 DNA copies/mm^3 among coinfected individuals with HIV-HTLV-1

Parameter	adjusted OR	95% (confidence interval)	p-value
**Use of DTG**	0.12	0.00 -0.44	0.004

Propensity score was used as a covariate in the model.
Variables used to calculate the propensity score: age, time of use of antiretroviral therapy and CD4+.

Bootstrap results are based on 1000 bootstrap samples

DTG: Dolutegravir, OR: odds ratio

Supplemental Table 5. Logistic regression using bootstrap resampling was performed to tested association between use of DTG and proviral load >50 DNA copies/mm^3 among coinfected individuals with HIV-HTLV-1

Parameter	adjusted OR	95% (confidence interval)	p-value
**Use of DTG**	0.20	0.04 - 0.70	0.008

Propensity score was used as a covariate in the model.
Variables used to calculate the propensity score: age, time of use of antiretroviral therapy and CD4+.

Bootstrap results are based on 1000 bootstrap samples

DTG: Dolutegravir, OR: odds ratio
